# Enhancing breast cancer treatment: mesoporous dopamine nanoparticles in synergy with chrysin for photothermal therapy

**DOI:** 10.3389/fonc.2024.1427858

**Published:** 2024-07-09

**Authors:** Jing Zhu, Heng Zhang, Haomiao Lan, Bing Bi, Xianfeng Peng, Dandan Li, Haili Wang, Ke Zhu, Fuqiang Shao, Minggang Yin

**Affiliations:** ^1^ Health Management Center, Zigong First People’s Hospital, Zigong, China; ^2^ Medical Imaging Center, Dazhou Central Hospital, Dazhou, China; ^3^ Center for Precision Health, School of Medical and Health Science, Edith Cowan University, Perth, WA, Australia; ^4^ Department of Thyroid and Breast Surgery, Zigong First People’s Hospital, Zigong, China; ^5^ Department of Nuclear Medicine, Zigong First People’s Hospital, Zigong, China; ^6^ Department of Clinical Laboratory, Zigong First People’s Hospital, Zigong, China

**Keywords:** breast cancer, chrysin, mesoporous dopamine, photothermal therapy, synergistic therapy

## Abstract

**Introduction:**

Breast cancer is one of the most prevalent cancers, primarily affecting women. Among its subtypes, estrogen receptor-positive (ER^+^) breast cancer is particularly common. Inhibiting estrogen's effects is crucial for treating ER^+^ breast cancer, but current therapies often have significant side effects and limitations. Chrysin, a natural flavonoid, has shown potential in reducing estrogen receptor expression, but its poor water solubility hampers clinical application. This study explores the use of mesoporous dopamine nanoparticles (mPDA) to enhance the delivery and efficacy of Chrysin, combined with photothermal therapy (PTT), for breast cancer treatment.

**Methods:**

Chrysin-loaded mPDA nanoparticles (Chrysin@mPDA) were synthesized and characterized for their morphology, drug-loading efficiency, stability, and photothermal properties. Network pharmacology was used to predict Chrysin's mechanisms in breast cancer, which were validated through gene expression analysis in cell experiments. The therapeutic efficacy of Chrysin@mPDA with and without PTT was evaluated in a mouse model of breast cancer, with tumor volume and weight measured. Immunohistochemical analysis was conducted to assess estrogen receptor expression and immune cell infiltration in tumor tissues.

**Results:**

Chrysin@mPDA nanoparticles demonstrated a high drug-loading capacity and excellent stability. Photothermal studies confirmed the nanoparticles' ability to generate heat upon laser exposure, significantly enhancing Chrysin release in acidic conditions with laser irradiation. Network pharmacology identified key target genes affected by Chrysin, including ESR1, BRCA1, CTNNB1, and BAX, which were validated through qPCR. *In vivo*, the combination of Chrysin@mPDA and PTT significantly reduced tumor volume and weight, decreased estrogen receptor-positive cells, and increased infiltration of CD3^+^CD4^+^ and CD3^+^CD8^+^ T cells in tumor tissues.

**Discussion:**

The study highlights the potential of Chrysin-loaded mPDA nanoparticles combined with PTT as an effective strategy for breast cancer treatment. This approach addresses the limitations of Chrysin's solubility and enhances its therapeutic efficacy through synergistic mechanisms. The dual action of Chrysin in modulating gene expression and PTT in inducing localized hyperthermia and immune response suggests a promising avenue for improved breast cancer prognosis and reduced recurrence.

## 1 Introduction

Breast cancer is one of the most common cancers, predominantly affecting women but occasionally occurring in men (1). Based on both molecular and histological evidences, breast cancer could be categorized into three groups, breast cancer expressing hormone receptor: estrogen receptor (ER^+^) or progesterone receptor (PR^+^); breast cancer expressing human epidermal receptor 2 (HER2^+^) and triple-negative breast cancer (TNBC) (ER^−^, PR^−^, HER2^−^) (2). Among the various subtypes of breast cancer, estrogen receptor-positive breast cancer (ER+ breast cancer) stands out as one of the most prevalent (1, 3). This subtype is characterized by the presence of estrogen receptors on the cancer cells, rendering them sensitive to the stimulatory effects of estrogen (1, 4).

In the context of breast cancer, inhibiting estrogen holds significant therapeutic significance (5). This commonly achieved through competitive inhibition of estrogen binding to tumor estrogen receptors, or down-regulating estrogen expression in the body, and reducing the expression of estrogen receptors on tumors (6–9). By either suppressing estrogen production or disrupting its interaction with cancer cells, the growth and proliferation of this subtype of breast cancer can be effectively controlled. Inhibiting estrogen halts the progression of ER^+^ breast cancer, as estrogen plays a pivotal role in promoting the growth of cancer cells (10). Clinical evidence has established that estrogen inhibition can enhance the prognosis of breast cancer, ultimately improving both survival rates and the quality of life for patients (5, 10). Moreover, estrogen inhibition can serve as a preventive measure against disease recurrence. It is typically integrated into a comprehensive treatment strategy, in conjunction with surgical interventions, radiotherapy, chemotherapy, and other therapeutic modalities, to augment treatment effectiveness (1, 11).

Despite the remarkable efficacy of estrogen inhibition in treating ER^+^ breast cancer, non-selective inhibition of estrogen always with a set of side effects and limitations, including reductions in bone density and the occurrence of hot flashes. Furthermore, estrogen inhibition often necessitates long-term or even lifelong treatment to sustain a stable disease state and reduce the risk of recurrence (1, 4).

Chrysin is a natural flavonoid found abundantly in plants like blue passion flowers, citrus fruits, and honey. Known for its bioactive properties, it has been an integral part of herbal remedies and traditional Chinese medicine, aimed at enhancing health and treating diverse ailments (12). In this study, we analyzed the mechanism of the possible action of Chrysin on breast cancer through network pharmacology, and found that Chrysin may have a unique anti-breast cancer effect by reducing the expression of estrogen receptor in breast cancer, and verified it through cell experiments and animal experiments.

If chrysin is applied to breast cancer cells to diminish the expression of the estrogen receptor, it can also effectively obstruct estrogen’s influence on these cells. Successfully translating this approach into clinical practice could serve as an alternative therapy to ovarian ablation for estrogen inhibition, potentially enhancing breast cancer prognosis and elevating patients’ quality of life.

Despite its benefits, the poor water solubility of Chrysin significantly hampers its clinical application. Nevertheless, the progression in nanotechnology, utilizing nano-carriers to enhance solubility and delivery, is forging innovative avenues for Chrysin’s deployment in cancer therapy and other medical fields. Concurrently, the unique properties of these nanoparticles can be leveraged to facilitate coordinated treatment strategies (13).

The present study focuses on the application of mesoporous dopamine nanoparticles (mPDA) based photothermal therapy (PTT) for breast cancer, concurrently utilizing mPDA as a carrier for the targeted delivery of chrysin to breast cancer tissues. Chrysin possesses the capacity to suppress estrogen receptor expression in breast cancer cells, thus thwarting the action of estrogen on breast cancer. This downregulation of estrogen receptor expression offers the potential to mitigate the side effects associated with conventional estrogen-blocking therapies. In addition, our study also found that PTT therapy significantly upregulates breast cancer infiltrating immune cells, which is not achieved by chrysin therapy alone. The number of infiltrated immune cells and the composition of them in the immune microenvironment can determine the outcome of breast cancer therapy. It is reported that a high tumor-infiltrating lymphocytes ratio is associated with better prognosis in breast cancer patients (14, 15). The **Schematic Figure** provides an overview of this study.

**Schematic Figure:**
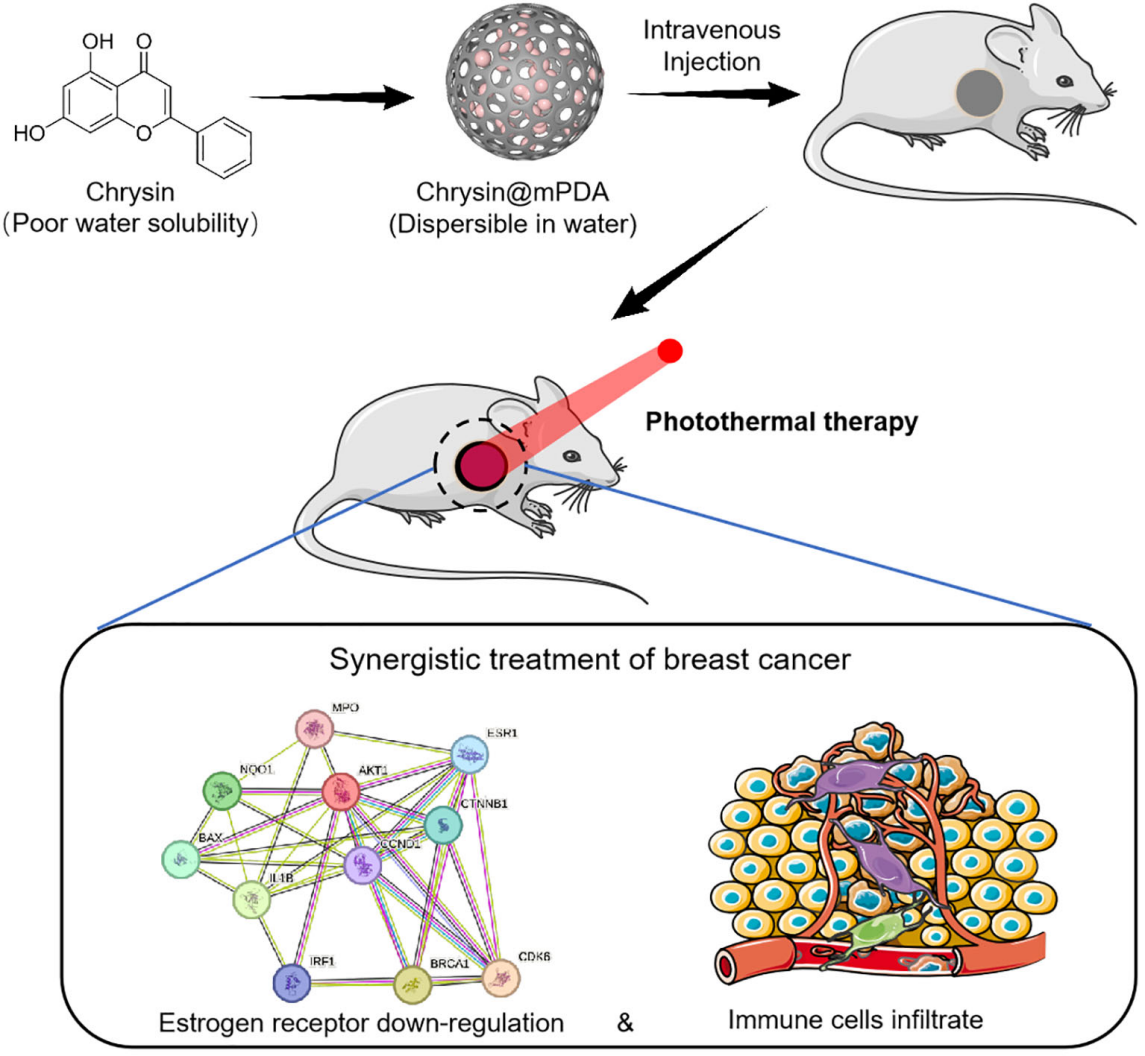
Schematic representation of the synergistic treatment of breast cancer using Chrysin and photothermal therapy (PTT). Chrysin, with poor water solubility, is loaded onto mPDA nanoparticles (Chrysin@mPDA) to improve dispersibility in water. After intravenous injection, the nanoparticles accumulate at the tumor site, where PTT is applied. This combination leads to estrogen receptor down-regulation and increased immune cell infiltration, enhancing the therapeutic efficacy against breast cancer.

## 2 Results

### 2.1 Characterization of chrysin-loaded mPDA nanoparticles

**Figure 1:**
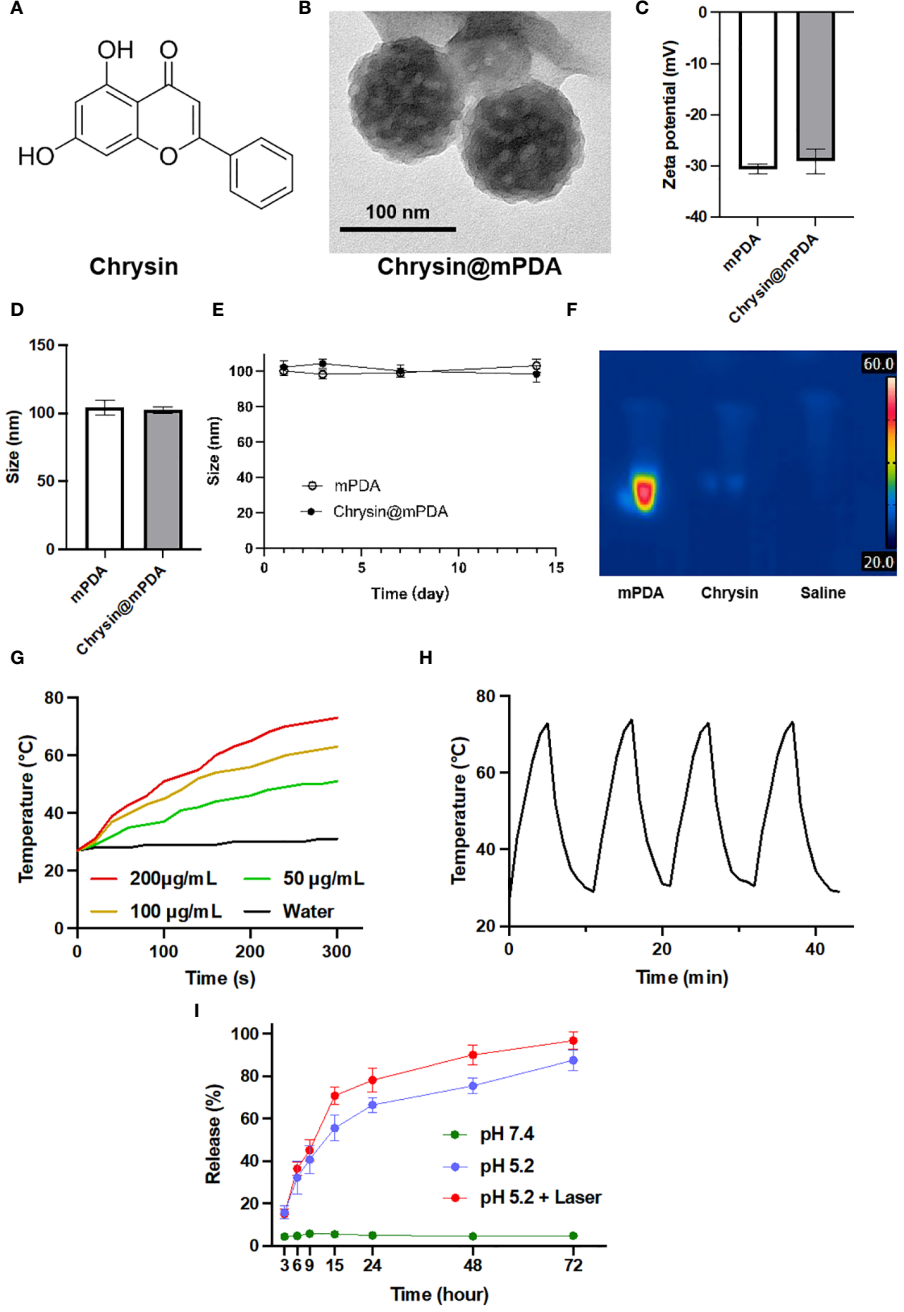
Characterization of Chrysin-Loaded mPDA Nanoparticles. **(A)** Chemical structure of Chrysin used in this study. **(B)** Transmission electron microscopy (TEM) image of Chrysin-loaded mesoporous polydopamine (mPDA) nanoparticles (Chrysin@mPDA). Scale bar: 100 nm. **(C)** Zeta potential measurements of mPDA and Chrysin@mPDA nanoparticles. **(D)** Size distribution of mPDA and Chrysin@mPDA nanoparticles. **(E)** Stability of mPDA and Chrysin@mPDA nanoparticles over a 14-day period. **(F)** Thermal imaging of mPDA, Chrysin, and saline under 808 nm laser irradiation. **(G)** Temperature change of mPDA dispersions with different concentrations (200, 100, 50 μg/mL) and water under 808 nm laser irradiation. **(H)** Photothermal stability of mPDA nanoparticles. **(I)** Drug release profiles of Chrysin@mPDA under different conditions. All bars represent as means ± SD (n = 3).

**Figure 1A** illustrates the molecular structure of Chrysin used in our study (12). **Figure 1B** showcases the morphology of Chrysin-loaded mesoporous dopamine (mPDA) nanoparticles (Chrysin@mPDA) prepared in our research. Compared to traditional PDA nanoparticles, mPDA nanoparticles exhibit a higher specific surface area and enhanced drug-loading capacity (16). The drug loading efficiency was quantified at 48.3% ± 1.4%, and drug encapsulation efficiency was 73.31 ± 2.1%. Furthermore, we measured the zeta potential and nanoparticle diameter of Chrysin@mPDA and mPDA. Notably, Chrysin loading had no significant impact on the zeta potential and nanoparticle size of mPDA (**Figures 1C, D**, all p > 0.05). The particle size remained at approximately 100 nm. It is worth noting that nanoparticles of this size are reported to exhibit excellent enhanced permeability and retention (EPR) effects in tumor tissues, allowing for effective accumulation in tumor sites (17–19). We also investigated the changes in nanoparticle size for mPDA and Chrysin@mPDA over two weeks. Nanoparticles was dispersed in phosphate buffered saline (PBS) and maintained under continuous incubation at 37°C, the results indicated that mPDA displayed good stability, with no significant aggregation or degradation affecting the particle size (**Figure 1E**).

In this study, mPDA will be utilized for PTT of tumor tissues. Therefore, we investigated the *in vitro* photothermal performance of mPDA. The results showed that the rate of temperature increase in the liquid upon exposure to 808 nm laser light accelerated with increasing mPDA concentration. Deionized water without mPDA exhibited a very slow temperature increase under 808 nm laser illumination, with the temperature remaining below 30°C during 5 minutes of exposure. In contrast, a nanoparticle dispersion containing 200 μg/mL of mPDA reached a temperature of 73°C after 5 minutes of laser exposure (**Figures 1F, G**). We also tested the effect of repeated photothermal cycles on mPDA’s photothermal performance. The results revealed that after three cycles of 808 nm laser exposure, mPDA continued to exhibit excellent photothermal capabilities in the fourth cycle (**Figure 1H**).

In addition, we evaluated the drug release rate of Chrysin@mPDA (**Figure 1I**). Given the typically acidic tumor microenvironment and the application of tumor PTT in our study, we divided the experiments into three groups, and the results illustrate the sustained release profiles of mPDA-loaded Chrysin under three different conditions: At pH 7.4, there was minimal change in the release of Chrysin, maintaining close to 5% throughout the entire duration. Under pH 5.2, the release of Chrysin gradually increased over time, reaching approximately 50% by around 15 hours. For the condition at pH 5.2 with laser exposure, at the 10-hour mark, the solution was exposed to an 808 nm near-infrared laser for 5 minutes, causing the release rate to rapidly climb to about 70% within the first 15 hours. It then continued to slowly increase, reaching nearly 90% by 60 hours.

### 2.2 The effects of chrysin in breast cancer and network pharmacy analysis

Chrysin, a natural flavonoid compound, is abundant in various plants such as Scutellaria baicalensis and Bupleurum falcatum. It boasts a millennia-old history of utilization in traditional Chinese medicine (12, 20, 21). In the realm of traditional Chinese medicine, the treatment mechanisms of natural compounds are often intricate, involving complex networks when applied to patients (22, 23). Confirming the primary mechanism by which Chrysin addresses breast cancer through quantitative animal experiments can be a formidable challenge. Network pharmacology emerges as an invaluable tool for predicting the mechanisms, toxicity, and metabolic characteristics of traditional Chinese medicines (24). Consequently, we embarked on a network pharmacological analysis to unveil the workings of Chrysin in the context of breast cancer.

**Figure 2:**
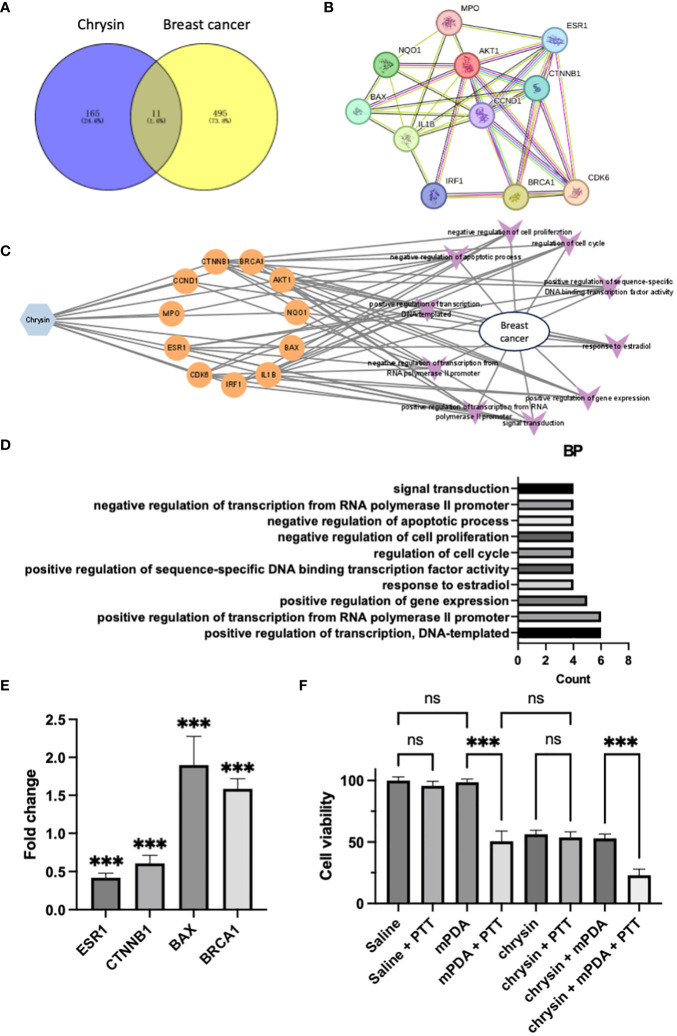
The Effects of Chrysin in Breast Cancer and Network Pharmacology Analysis. **(A)** Venn diagram showing the overlap between target genes of Chrysin and breast cancer-related genes. **(B)** Protein-protein interaction (PPI) network of the 11 core genes identified in the analysis. **(C)** Drug-target pathway network illustrating the interaction involved in breast cancer. **(D)** Gene ontology (GO) enrichment analysis of the top 10 significantly enriched biological processes (BP) with a significance level of P < 0.01. Key terms related to breast cancer treatment. **(E)** Gene expression analysis (qPCR) of ESR1, CTNNB1, BAX, and BRCA1 in MDA231 cells treated with Chrysin. **(F)** Cell viability assay of MDA231 cells under different treatment conditions. All bars represent as means ± SD (n = 3), and ***p < 0.001, ns p ≥ 0.05.

During our investigation, we scrutinized 178 target genes linked to Chrysin and 510 target genes associated with breast cancer. This meticulous analysis led us to the identification of 11 specific genes that we deem vital in the breast cancer treatment, visually presented in a Venn diagram (**Figure 2A**). Taking a deeper dive into our analysis, we employed Cytoscape v3.7.2 software to explore the topological features of the protein-protein interaction (PPI) network. Here, we pinpointed 11 core genes (ESR1, BRCA1, CTNNB1, BAX, CCND1, AKT1, NQO1, IL-1B, MPO, IRF1, CDK6) and fashioned a PPI core network comprising these 11 nodes, as illustrated in **Figure 2B**. It is noteworthy that ESR1, BRCA1, CTNNB1, and BAX occupied central positions within the PPI network. To further explore the roles of these 11 shared target genes in breast cancer, we conducted KEGG pathway enrichment analysis with a significance threshold of P < 0.01. Using the enriched KEGG pathways, we constructed a “drug-target-pathway” network as depicted in **Figure 2C**. This network provided insights into the comprehensive nature of chrysin’s breast cancer treatment, involving multiple components, multiple targets, and multiple pathways.

To reveal the biological characteristics of these 11 shared target genes, we carried out gene ontology (GO) enrichment analysis using the Metascape tool. Our selection criteria included a significance level of P < 0.01, minimum enrichment >1.5, and a minimum overlap of 3. **Figure 2D** showcases the top 10 significantly enriched terms within the context of biological processes (BP). After excluding terms not directly related to breast cancer treatment, we observed that “response to estradiol” and “regulation of cell cycle” emerged as the prominent terms, providing further evidence that chrysin’s therapeutic mechanism in breast cancer revolves around interrupting estrogen-related effects.

In our experiment, the application of chrysin to MDA231 cells, followed by qPCR gene expression analysis, yielded significant outcomes. Specifically, we observed a pronounced downregulation of ESR1 gene (estrogen receptor gene) expression, accompanied by a significant decrease in the expression of the oncogene CTNNB1. Simultaneously, we noted a substantial upregulation in the expression of the anti-oncogene genes BAX and BRCA1 (**Figure 2E**, all P < 0.05 compared with control group), and the findings align with the results presented in **Figure 2D**.

And we also validated the cytotoxicity of Chrysin in combination with PTT on breast cancer cells (**Figure 2F**). When MDA231 cells were incubated in PBS without mPDA (dispersed in saline), PTT treatment did not significantly affect cell viability compared to the Saline group. Similarly, the co-incubation of MDA231 cells with mPDA-containing PBS had no significant impact on cell viability. However, when cells were simultaneously exposed to mPDA-containing PBS and subjected to PTT treatment, cell viability decreased significantly. Treatment with Chrysin alone resulted in a notable reduction in cell viability. Notably, the cell viability in the Chrysin + PTT group and Chrysin + mPDA group did not exhibit significant differences compared to the Chrysin-only group, while the Chrysin + mPDA + PTT group showed the lowest cell viability among all groups. These results underscore the potential of Chrysin in combination with photothermal therapy for synergistic breast cancer management.

### 2.3 Breast cancer treatment

**Figure 3:**
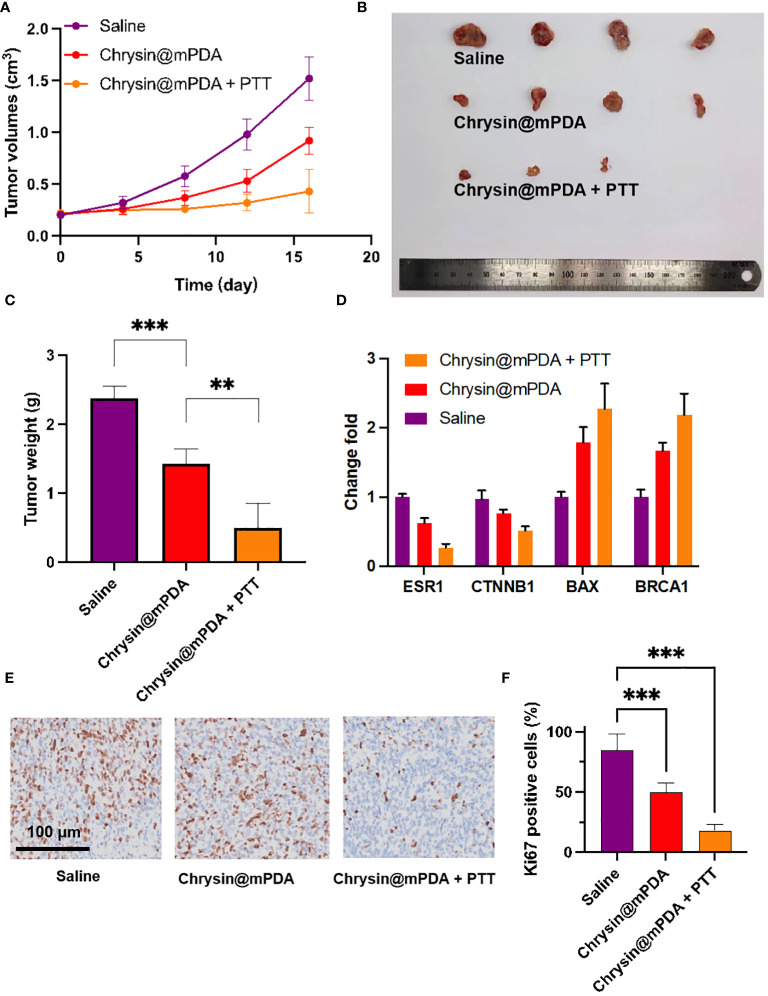
Breast Cancer Treatment with Chrysin@mPDA Nanoparticles PTT. **(A)** Tumor volume measurements in tumor-bearing mice treated with Saline, Chrysin@mPDA, and Chrysin@mPDA + PTT over 16 days. **(B)** Representative images of tumors excised from mice in the different treatment groups on the 16th day. **(C)** Average tumor weights from each treatment group. **(D)** qPCR analysis of ESR1, BRCA1, CTNNB1, and BAX expression in tumor tissues from different treatment groups. **(E)** Immunohistochemical staining of tumor tissues for Ki67, a marker of cell proliferation. **(F)** Quantification of Ki67 staining levels in the tumor tissues. All bars represent as means ± SD (n = 3), and **p < 0.01, ***p < 0.001.

All of the drugs were injected into the mice through the tail vein, and PTT was conducted 24 hours after the injection of nanoparticles. Following laser irradiation, the tumor sizes and weights in the tumor-bearing mice were continually monitored. **Figure 3A** illustrates the tumor volume of the saline group, which steadily increased, reaching 1.52 ± 0.21 cm³ on the 16th day. In contrast, the Chrysin@mPDA and Chrysin@mPDA + PTT groups exhibited a significant decrease in tumor volume. By the 16th day, the mice in the Chrysin@mPDA + PTT group had the smallest tumor volume, measuring 0.43 ± 0.21 cm³, indicating the most effective treatment outcomes. This trend was consistent with the representative images and average tumor weights displayed in **Figures 3B, C**. It is evident that Chrysin@mPDA played a role in suppressing breast cancer growth, and the combination of Chrysin@mPDA with PTT demonstrated synergistic effects in breast cancer treatment.

To further validate these findings, we conducted qPCR analysis to assess the expression of ESR1, BRCA1, CTNNB1, and BAX in tumor tissue. The results were consistent with the data presented in **Figures 2B–F**, demonstrating that Chrysin could regulate the expression of ESR1, oncogenes, and tumor suppressor genes within the tumor (**Figure 3D**). ESR1, BRCA1, CTNNB1, and BAX was associated with breast cancer cells proliferation, we further employed Ki67 immunohistochemical staining to evaluate tumor cell proliferation (**Figure 3E**). The levels of Ki67 staining in the tumor tissues of the Chrysin@mPDA + PTT group were lower compared to the Chrysin@mPDA group (**Figure 3F**). These findings confirm that the Chrysin@mPDA + PTT group achieved the most effective tumor treatment. Our study highlights the potential of Chrysin-loaded mPDA nanoparticles in combination with PTT as an effective treatment strategy for breast cancer. This approach leads to a significant reduction in tumor volume and weight, accompanied by the regulation of key genes associated with breast cancer cell proliferation. The combination of Chrysin and PTT holds promise as a synergistic and potent therapy for breast cancer management, offering new avenues for future research and clinical applications.

### 2.4 Analysis of possible mechanisms of chrysin and PTT coordination in the treatment of breast cancer

**Figure 4:**
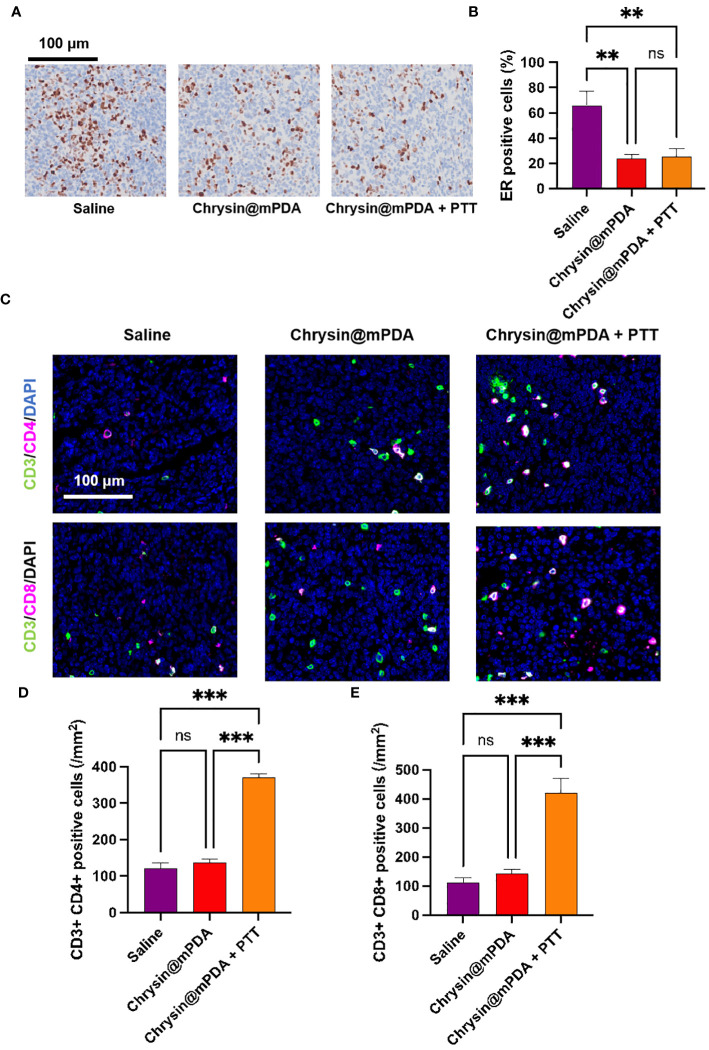
Analysis of Mechanisms Behind Chrysin and PTT Coordination in Breast Cancer Treatment. **(A)** Immunohistochemical staining images of ESR-positive cells in tumor tissues from different treatment groups. **(B)** Quantitative analysis of ESR-positive cells in tumor tissues. **(C)** Immunofluorescence staining images of CD3^+^CD4^+^ helper T cells and CD3^+^CD8^+^ cytotoxic T cells in tumor tissues from different treatment groups. **(D)** Quantitative analysis of CD3^+^CD4^+^ helper T cells in tumor tissues. **(E)** Quantitative analysis of CD3^+^CD8^+^ cytotoxic T cells in tumor tissues, showing a significant increase in the Chrysin@mPDA + PTT group compared to other groups. All bars represent as means ± SD (n = 6), and **p < 0.01, ***p < 0.001, ns p ≥ 0.05.

We had previously demonstrated Chrysin’s ability to reduce ESR expression. To further validate this, we conducted immunohistochemical analysis of the number of ESR-positive cells in tumor tissue, with representative images displayed in **Figure 4A**. Through quantitative analysis (**Figure 4B**), we confirmed that treatment with Chrysin@mPDA significantly reduced the number of ESR-positive cells in tumor tissue, and the Chrysin@mPDA + PTT group showed no further decrease or increase in ESR-positive cells, demonstrating the unique role of chrysin. The reduction in the number of ESR-positive cells indicates a decreased sensitivity of the tumor tissue to estrogen. This reduction in estrogen sensitivity is beneficial in reducing the ability of estrogen to stimulate the growth of breast cancer tumor tissue.

It has been reported that PTT can enhance the immune response in cancer treatment (25–27). To further investigate the potential synergistic benefits of Chrysin@mPDA + PTT treatment for breast cancer, we conducted an analysis of the subpopulations of CD3^+^CD4^+^ helper and CD3^+^CD8^+^ cytotoxic T cells. The immunomodulatory effects of each treatment group were assessed using tissue immunofluorescence assays, with representative figures presented in **Figure 4C**.

In the Chrysin@mPDA group, there was no significant difference in the number of CD3^+^CD4^+^ and CD3^+^CD8^+^ T cells in the tumor tissues. However, in the Chrysin@mPDA + PTT group, the results were markedly different. The number of CD3^+^CD4^+^ and CD3^+^CD8^+^ cells in breast cancer tissue was significantly enhanced, indicating a robust antitumor immune response (**Figures 4D, E**). These findings suggest that in the Chrysin@mPDA + PTT therapy, PTT plays a crucial role in inducing tumor immunity. This implies that the synergistic treatment of Chrysin@mPDA + PTT not only overcomes the limitation of Chrysin therapy in solely inhibiting tumor cell proliferation but also provides a systemic antitumor effect.

## 3 Discussion

This study ventures further into the innovative approaches for enhancing the bioavailability and efficacy of Chrysin. Chrysin, a flavonoid found in numerous plants, has been utilized in traditional Chinese medicine for thousands of years due to its anti-inflammatory, anti-oxidant, and anti-cancer properties (21). The application of network pharmacology in this research sheds light on Chrysin’s complex interaction networks within the biological system, particularly its effects on breast cancer.

One of the main hurdles in utilizing Chrysin effectively for clinical purposes is its low solubility in water, which greatly limits its bioavailability and therapeutic efficiency (28). Recent advancements in nanotechnology have presented new opportunities to circumvent these limitations. The development and application of various nano-carriers have revolutionized drug delivery systems, allowing for increased solubility, stability, and targeted delivery of therapeutic agents. In this context, the study explored the use of melanin-like polydopamine (mPDA) nanoparticles as a novel delivery system for Chrysin. The mPDA nanoparticles were chosen for their biocompatibility, ease of functionalization, and their intrinsic photothermal conversion properties, making them an ideal carrier for both Chrysin and PTT (28, 29).

By encapsulating Chrysin within mPDA nanoparticles, we achieved a significantly high drug loading capacity, which not only addressed the issue of Chrysin’s solubility but also enabled effective concentration of the drug at the tumor site (28, 30). The integration of Chrysin and PTT, facilitated by mPDA nanoparticles, represents a synergistic strategy that leverages the strengths of each component. Chrysin acts on the cellular level, modulating gene expression to inhibit oncogenes and promote anti-oncogenes, thereby disrupting cancer cell proliferation and survival pathways. Concurrently, PTT generates localized hyperthermia that induces direct thermal damage to the tumor cells. This dual modality not only targets the tumor cells more effectively but also stimulates the immune system, as evidenced by the significant increase in CD3^+^CD4^+^ and CD3^+^CD8^+^ T cell populations within the tumor microenvironment. This enhancement of the antitumor immune response indicates a potential for not just local tumor control but also systemic immunomodulatory effects.

Further, the study also delves into the implications of such treatment strategies on the broader scope of cancer therapy. The ability to induce a systemic immune response suggests a potential for preventing metastasis and recurrence, two major challenges in breast cancer management. The findings from this research underscore the importance of an integrated approach that combines traditional pharmacology with modern nanotechnology and immunotherapy techniques.

In conclusion, the Chrysin@mPDA + PTT strategy introduces a highly promising avenue for breast cancer treatment, overcoming previous limitations through innovative drug delivery systems and enhancing therapeutic outcomes through synergistic mechanisms. Future studies could focus on optimizing the nanoparticle formulation for even higher drug loading capacities, exploring the use of combination therapies with other anticancer agents, and conducting clinical trials to validate the efficacy and safety of this approach in human patients. This research not only contributes to the field of cancer therapy but also exemplifies the potential of combining traditional medicine with modern technology to address complex health challenges.

## 4 Conclusion

In this research, we developed mPDA nanoparticles for the delivery of Chrysin, effectively overcoming its poor water solubility and facilitating its application in cancer treatment. Leveraging the light-absorbing capacity of mPDA nanoparticles, we employed PTT therapy combined with Chrysin, revealing a powerful synergy in tumor treatment. Our study has illuminated the multifaceted mechanisms by which Chrysin operates in breast cancer therapy, emphasizing the critical role of PTT in inducing tumor immunity. This synergistic approach not only surmounts the limitations of Chrysin therapy in solely inhibiting tumor cell proliferation but also provides a comprehensive and systemic antitumor effect. These findings not only broaden the horizons of breast cancer treatment but also underscore the potential of combined Chrysin@mPDA + PTT therapy in the battle against this challenging disease. However, we need to point out the shortcomings of our study, that is, it will take a long time to conduct a comprehensive and detailed evaluation of the safety of mPDA for clinical use.

## 5 Experimental

*Materials:* Chrysin was sourced from Yuanye in China. Dopamine was obtained from Sigma-Aldrich in the USA. PBS was purchased from Gibco Life Technologies in Gaithersburg, MD, USA. CCK-8 kits were acquired from Boster Biotechnology in Wuhan, China. CTAB was procured from Sigma-Aldrich in the USA.

*Synthesis of Nanoparticles and Characterization:* The synthesis of mPDA followed a previously established method. In brief, 250 mg of dopamine and 500 mg of pluronic F-127 were dissolved in a 1:1 water and ethanol mixture (50 mL) and vigorously stirred to achieve a clear solution. Subsequently, 1,3,5-trimethylbenzene was slowly added dropwise while stirring at 500 rpm to create a nanoemulsion system. After 30 minutes, 2.5 mL of NH4OH was added drop by drop to initiate the self-polymerization of dopamine oligomers. After a 2-hour reaction, the freshly synthesized mPDA was centrifuged and washed twice with acetone. The resulting mPDA was then washed three times with a 1:1 water and ethanol solution. mPDA and Chrysin@mPDA were characterized using transmission electron microscopy (TEM, Hitachi, Japan) and dynamic light scattering (DLS) analysis (Malvern Instruments Ltd, Worcestershire, UK). To assess the stability of mPDA and Chrysin@mPDA, nanoparticles was dispersed in PBS and maintained under continuous incubation at 37°C, the hydrodynamic diameters were monitored for 14 days using a DLS system. Solutions of mPDA at different concentrations (50, 100, and 200 μg mL−1) in saline were prepared. To evaluate the photothermal properties of the nanoparticles, they were dispersed in a quartz cuvette and exposed to an 808 nm laser at a constant power (0.70 W cm−2) for 200 seconds. Temperature changes and thermal images were recorded at various time points. The encapsulation efficiency (EE) of Chrysin in mPDA was determined by measuring the amount of unencapsulated drug in the supernatant after centrifugation. The concentration of Chrysin was quantified using liquid chromatography-mass spectrometry, and EE was calculated using the formula: EE = [Weight of loaded drug/Weight of initial drug input] × 100%. After freeze-drying the nanoparticles to obtain nanoparticle quality, the drug loading capacity (LC) was calculated using the formula: LC = [Weight of loaded drug/Weight of drug-loaded nanoparticles] × 100%.

*Network pharmacology analysis*: The targets related to breast cancer were obtained from GeneCards (http://www.genecards.org), Drugbank (https://go.drugbank.com/), OMIM (https://www.omim.org/), TTD (https://db.idrblab.net/ttd/); The targets related to Chrysin were obtained from CTD (http://ctdbase.org/voc.go?type=chem) and TCMSP (http://lsp.nwu.edu.cn/tcmsp). The intersection genes of the Chrysin and breast cancer were also obtained Venny 2.1. Protein-protein interaction (PPI) network was constructed using the STRING online tool (https://string-db.org) with a confidence score above 0.9. The “compound-target-biological processes” (C-T-B) network was constructed using Cytoscape v3.7.2 software. These overlapping genes were imported into the DAVID online platform (https://david.ncifcrf.gov) for KEGG enrichment analysis.

*In vitro experimental*: MDA231 cells were cultured in DMEM supplemented with 10% fetal bovine serum (FBS), 100 U/mL penicillin, and 100 μg/mL streptomycin at 37°C in a humidified atmosphere with 5% CO_2_. Cells were seeded in 96-well plates at a density of 5 × 10^3 cells per well and allowed to adhere overnight. To verify the network pharmacological analysis, cultured MDA231 cells were added with a concentration of 100 μM Chrysin, incubated for 12 hours, and the cells were collected for RNA extraction using the TRIzol method. RNA from each sample was reverse-transcribed into cDNA using the Reverse Transcription kit (Toyobo, Japan). The qRT-PCR was performed with the SYBR^®^ Premix Ex Taq ™ II Kit (Toyobo, Japan). Primers were designed based on the cDNA sequence in NCBI. GAPDH mRNA was used as an internal reference for detection. The qRT-PCR reaction system includes: 2 μL of cDNA, 2 μL of primer (1 μmol/L), 10 μL of SYBR green dye (2×), and 6 μL of Nuclease-Free H_2_O. The total reaction system is 20 μL. Reaction conditions: 95°C for 30 s, 95°C for 3 s, 55°C for 30 s, 72°C for 30 s, 95°C for 60 s, 55°C for 30 s, and 95°C for 30 s. 42 amplification cycles were performed. The PCR was confirmed by the product melting curve, and the Ct values of the target gene and GAPDH of the sample were obtained based on the PCR curve. The relative expression level for target genes were performed with a value of 2^-ΔΔCt^. The cytotoxicity of different treatments on MDA231 cells (Chrysin concentration is 100 μM, and mPDA is 100 μg/mL), with or without exposure to an 808 nm laser (0.70 W cm^−2^ for 200 seconds), was assessed using a CCK-8 assay.

*Tumor-Bearing Mouse Models:* MDA231 cells (1 × 106) suspended in 100 μL of PBS were subcutaneously injected into the right upper limb of 6-week-old female C57BL/6 mice sourced from Beijing HFK Bioscience Co., Ltd, China, when the tumor size reached approximately 5 mm. For the breast cancer mouse models, 6-week-old C57BL/6 mice underwent laparotomy to expose their cecum, and then MDA231 cells (1 × 106) suspended in 50 μL of PBS were injected at the subcutaneous site of the leg.

*Treatment To Breast Cancer Mouse Models:* MDA231 tumor-bearing mice were allocated randomly into three groups, each consisting of four mice. The groups received the following treatments: normal saline (saline group), Chrysin@mPDA at a dose of 2 mg kg−1, Chrysin@mPDA at a dose of 2 mg kg−1 with laser (Chrysin@mPDA + PPT group). All of the drugs were injected into the mice through the tail vein. After 24 hours of injection, the Chrysin@mPDA + PPT group receiving laser treatment were anesthetized using 2% isoflurane, and their tumor sites were subjected to 1.4 W·cm−2 808 nm laser irradiation for 5 minutes. The changes in temperature were monitored using a thermal imaging camera. Subsequently, the tumor sizes and body weights of the mice were measured to 16 days. On the 16th day, all the mice were euthanized. The tumor tissues were collected, weighed, photographed, and preserved for further histological examinations. Immunohistochemistry was conducted to evaluate the Ki67 positive cells, ESR positive cells, CD3^+^CD4^+^ and CD3^+^CD8^+^ positive T cells.

*Statistical analyses:* The data is presented as mean ± standard deviation (SD). Group comparisons were assessed using the unpaired Student’s t-test. Statistical significance was defined as P < 0.05. Statistical analyses were performed utilizing GraphPad Prism version 8.0 software.

## Data availability statement

Publicly available datasets were analyzed in this study. These datasets can be accessed at the following sources: GeneCards (http://www.genecards.org), DrugBank (https://go.drugbank.com), OMIM (https://www.omim.org), the Therapeutic Target Database (TTD) (https://db.idrblab.net/ttd), the Comparative Toxicogenomics Database (CTD) (http://ctdbase.org/voc.go?type=chem), and the Traditional Chinese Medicine Systems Pharmacology Database and Analysis Platform (TCMSP) (https://old.tcmsp-e.com/tcmsp.php). For further inquiries, please contact the corresponding authors.

## Ethics statement

The animal study was approved by Ethical Review Committee and Laboratory Animal Welfare Committee of Zigong First People’s Hospital. The study was conducted in accordance with the local legislation and institutional requirements.

## Author contributions

JZ: Methodology, Writing – original draft. HZ: Data curation, Writing – original draft, Writing – review & editing. HL: Methodology, Writing – original draft. BB: Data curation, Writing – original draft. XP: Formal analysis, Investigation, Methodology, Software, Writing – original draft. DL: Formal analysis, Investigation, Methodology, Software, Writing – review & editing. HW: Formal analysis, Investigation, Writing – review & editing. KZ: Writing – original draft, Writing – review & editing. FS: Conceptualization, Funding acquisition, Investigation, Supervision, Writing – original draft, Writing – review & editing. MY: Conceptualization, Supervision, Writing – review & editing.
